# GPs’ and dentists’ experiences and expectations of interprofessional collaboration: findings from a qualitative study in Germany

**DOI:** 10.1186/s12913-017-2116-4

**Published:** 2017-03-07

**Authors:** Khira Sippli, Monika A. Rieger, Fabian Huettig

**Affiliations:** 1 Department of Prosthodontics, Centre for Dentistry, Oral Medicine, and Maxillofacial Surgery at the University Hospital Tuebingen, Eberhard-Karls-University Tuebingen, Osianderstr. 2-8, D-72076 Tübingen, Germany; 2 Institute of Occupational and Social Medicine and Health Services Research, University Hospital of Tuebingen, Wilhelmstr. 27, D-72074 Tübingen, Germany; 3 CoreFacility/Coordinating Center Health Services Research, Faculty of Medicine, Eberhard Karls University Tuebingen, Wilhelmstr. 27, D-72074 Tübingen, Germany

**Keywords:** Cooperative behaviour, Oral health, Systemic diseases, Non communicable diseases, Professional identity, Interprofessional collaboration, Periodontitis, Dentistry, Primary health care physicians, General practitioners

## Abstract

**Background:**

Against the background of well-described associations between oral and general health, collaboration between dentists and general practitioners (GP) is crucial to provide therapeutic and preventive patient care. However, in the German health system, GPs and dentists are organizationally separated, implying that interprofessional collaboration can only occur informally and on a voluntary basis. Given the scarce evidence of interprofessional collaboration between dentists and GPs, an explorative study was conducted. This paper outlines the findings of this study with regard to GPs’ and dentists’ experiences and expectations of interprofessional collaboration.

**Methods:**

Semi-structured interviews were conducted with GPs (*n* = 15) and dentists (*n* = 13) from three structurally different regions in Baden-Wurttemberg, Germany. The interview guide included questions on occasions, expectations and experiences of interprofessional collaboration. The interviews were transcribed verbatim and analysed using qualitative content analysis according to Mayring.

**Results:**

Both GPs and dentists reported perceived knowledge deficits of the other profession with regard to medication, particularly anticoagulants and bisphosphonates, as well as systemic and general respectively dental diseases. Expectations regarding the scope of collaboration diverge: whereas dentists were interested in extending collaboration, most GPs saw no need for collaboration.

**Conclusions:**

The perceived medical knowledge deficits of the other profession as well as divergent expectations concerning the scope of collaboration hinder profound and regular interprofessional collaboration between GPs and dentists. These perceived knowledge deficits may be rooted in the separate education of dentists and GPs in Germany. Fostering interprofessional education is a promising way to improve cooperation between GPs and dentists in the long term.

## Background

Evidence about the connection between oral health and systemic diseases has been discussed for decades and has increased in the past 10 years [1–3]. This evidence particularly addresses the interactions between periodontitis or remaining teeth and chronic, non-communicable diseases (diabetes, coronary heart disease, arteriosclerosis, dementia) [4–6]. To provide high-quality health care to patients, cooperation between general practitioners (GP) and dental practitioners (dentists, DP) is crucial. In Germany, health care and oral health care are organizationally separated, starting with the education of both types of professionals. Thus, administrative referrals between the two professions are not provided by the system, and cooperation between dentists and GPs is not formalized [7].

### Educational framework in Germany

Twenty-nine out of 34 medical faculties at public universities offer the study programme “Dental Medicine” (DM) in parallel with the study programme “Human Medicine” (HM).

HM encompasses all courses and studies needed for the state examination to become a physician. The construction of the six-year curriculum and the state exam in HM are established within the medical licensure regulations (Approbationsordnung für Ärzte, ÄAppO) [8]. These abstract directives provide medical faculties with a large scope of educational action. Commissioned by the conference of federal ministries of education, the Society of Medical Education in Germany (GMA) and the Council of Medical Faculties (MFT) recently developed a national, competence-based catalogue of learning objectives for undergraduate medical education (NKLM) [9].

This catalogue was launched in 2015 and is being implemented in the curricula [10]. Dental and oral health aspects are addressed in 40 of 2471 items (1.6% of all learning aims, competencies, diseases and reasons for consultation). These 40 items encompass the basics of teeth and tooth development as well as the investigation of the oropharynx and differential diagnosis of oro-facial pain. Dental medicine is neither a lecture series nor part of the medical state examination. After state examination, physicians must specialize in a four- to five-year programme (i.e., as a general practitioner) to treat patients without supervision in practice. These specializations are regulated by the medical council (chamber of physicians, Ärztekammer, ÄK). If specialized physicians (e.g., GPs, dermatologists) want to treat patients subsidized by the statutory health insurance (SHI), they need to apply to the Association of Statutory Health Insurance Physicians (Kassenärztliche Vereinigung, KV) to obtain a license.

Dental medicine is taught in the 29 dental schools at the previously mentioned universities with a medical faculty. The five-year curriculum ends with an additional half-year examination (state exam) and is regulated within the dentistry licensure regulations (Approbationsordnung für Zahnärzte, ZÄPrO) [11]. DM encompasses courses of the HM curriculum in the preclinical semesters (biochemistry, anatomy, physiology, physics, biology, chemistry). Most of these courses are specifically “designed” for dental students, or dental students are required to attend only some of the workshops. The extent of this measure differs between universities, but it implies that in most universities, dental and medical students learn together only occasionally and irregularly during preclinical courses. During the clinical curriculum, in addition to the subjects of dentistry, dental students attend lectures in dermatology, internal medicine, histological pathology, pharmacology, general surgery, microbiology, and clinical chemistry. These lectures are mandatory, and each is part of an oral exam during the state examination. However, these lectures are also specifically designed for dental students. Thus, dental students are not in contact with medical students.

Parallel to the HM, a task force established a competence-based catalogue of learning objectives for undergraduate dental education (NKLZ) [12]. This catalogue was launched in 2015 and is still being implemented in the curricula [10]. Among the 2199 items, the aspects of general medicine are addressed in three chapters including 70 reasons for consultation, 32 interactions and 131 relevant diseases (10.5% of all learning aims, competencies, diseases and reasons for consultation).

After the state examination, a dentist is allowed to work in private practice with only one restriction: dentists must work in a practice licensed by the Association of SHI Dentists (Kassenzahnärztliche Vereinigung, KZV) for 2 years to apply for their own license to treat patients with SHI subsidization. Dentists can voluntarily pass an additional specialization as an orthodontist and/or oral surgeon. Both specializations are regulated by the dental chamber in a three- to four-year programme combining work in dental practice and dental clinics.

### Framework of professionalism (daily practice) in Germany

As described above, after examination, dentists and physicians are regulated by separate organizations. First, the Chamber of Dentists (ZK, including oral surgeons, orthodontists) and the Chamber of Physicians (ÄK, covering all specialties) are responsible for professional rights, self-government of their professionals, including ethical considerations and licensing of specializations, and further educational programmes. This membership is mandatory. Second, the Association of Statutory Health Insurance Dentists (KZV) and the Association of Statutory Health Insurance Physicians (KV) regulate the settlement of treatments paid by the SHI between their licensed dentists/physicians and the SHIs. The KV and the KZV negotiate budgets for their members with the SHI and act as an interposed organization for the payment of medical/dental health care services.

Chambers and SHI Associations are organized as professional bodies at the federal and national levels; thus, there are two data pools and an administrative separation of oral health care and health care, with no transfer of information between these professional bodies. Consequently, dentists and physicians are unable to refer patients in an institutionalized way [7]. Nevertheless, both types of professionals are able to recommend that their patients see a dentist or GP, respectively (a type of assignment). Whereas referrals between physicians require a report about the findings, assignments between dentists and GPs are nonbinding.

Moreover, the separation of the chambers impedes an interdisciplinary discussion of the content of the further and continuing education of both professions.

### Research on interprofessional care of dentists and GPs

GPs are specialized physicians and (other than dentists) the most visited doctors in Germany [13]. Although numerous international studies address interprofessional collaboration between GPs and specialists [14–18], pharmacists or nurses [19–22], research has neglected cooperation between GPs and dentists. The abovementioned framework of daily practice hinders quantitative and effective health services research at the interface of these areas.

To our knowledge, only two qualitative studies have been conducted independently in Germany to explore this interface of health care [23, 24]. Both studies examined experiences of collaboration between dentists and GPs and how knowledge about the connection between oral health and systemic diseases is implemented in their daily routines. Both studies concluded that collaboration is limited to issues of anticoagulation and diabetes and is mostly accompanied by mutual criticism of patient management as well as knowledge deficits. Interestingly, both studies reported that cooperation works better between colleagues who are personally known to one another. In summary, cooperation is hindered mostly by insecurities about the other profession’s knowledge and daily practice.

### Objective of research

It remains necessary to comprehend the essentials of these mutual perceptions to improve collaboration [25]. Therefore, the existing interviews of Huettig et al. [23] were further analysed with regard to perceptions of the interviewees’ own professional role, expectations of the other professional group, and experiences with collaboration The results of this exploration are presented in this paper.

## Methods

### Study design and sample

The objective of the study was to explore GPs’ and dentists’ views and experiences regarding interprofessional collaboration. As this question has previously been neglected by research, a qualitative study design was chosen.

To account for potential differences in cooperation between GPs and dentists deriving from a difference in physician/dentist density in different regions (metropolis effect) [26, 27], participants were recruited from three kinds of structural regions: a city (Stuttgart), a metropolitan area (Reutlingen/Metzingen) and a rural area (district of Sigmaringen).

Five GPs and five dentists from each of these regions were recruited by telephone using contact addresses given by quality circles (a voluntary association of doctors that meets periodically to maintain high quality standards) as well as the physicians’ and dentists’ chamber of the district. Consideration was given only to general practitioners who did not specialize in specific patient groups (e.g., working as a paediatrician) and to dentists who did not specialize in oral/maxillofacial surgery, paediatric dentistry or orthodontics [23]. Compensation of 40 Euros was offered as an incentive for participation.

### Data collection and setting

On the basis of existing literature and according to the stepwise SPSS (Collect, Check, Sort, Subsume) method [28], an interview guide for the semi-structured interviews was developed. The interview guide contained six open questions to prompt narration as well as follow-up questions to enquire about topics (Table 1).

**Table 1:** The six consecutive leading questions of the interview guide

With regard to patient care, what are the reasons for you to contact a dentist/GP^a^ and how do you contact the dentist/GP^a^?
Which of your patients do you advise to see a dentist/GP^a^?
Which diseases could a dentist/GP^a^ possibly identify early and send the patient to you?
How have you experienced collaboration with dentists/GPs^a^?
In your opinion, which factors are most important for you have a fruitful collaboration with dentists/GPs^a^? In your view, what are the challenges to collaboration with dentists/GPs^a^?
What are your wishes for future collaborations with dentists and GPs?

^a^Question was modified depending on specialty of interviewee

In addition, sociodemographic and general information (e.g., gender, age, practice, routine, working experience) on the participants was collected using a form (see Table 2).

**Table 2:** Sample of participating GPs and dentists

Characteristic	GPs	Dentists
Sex	*N* = 15	*N* =13
Male	*N* = 10	*N* = 12
Female	*N* = 5	*N* = 1
Age (mean)	57 years	52 years
Number of years in practice (ø)	27 years	25 years
Size of practice team (ø)	1.33 doctors	1.85 doctors
Size of practice (ø)	3.5 rooms	3.5 rooms
Patients per day (ø)	55 patients/day	33 patients/day
Members in a quality circle	4	3
Rural area (letter L)	5	4
Medium-sized town (letter M)	6	5
City (letter G)	4	4

Qualitative semi-structured interviews were conducted by one interviewer (FH) with GPs (*n* = 15) and dentists (*N* = 13) between November 2013 and May 2014. The interviews occurred in the interviewee’s practice after office hours and lasted approximately 30 minutes each.

The interviews were transcribed verbatim by an external transcription service and pseudonymized.

### Data analysis

Data analysis began before all interviews were conducted so that the researchers could control for topic saturation [29]. Topic saturation occurred after the 20th interview. Thus, interviews were completed with only 28 of the 30 recruited participants.

Qualitative content analysis was conducted according to Mayring [30]. The categories were developed inductively from the material of the first eight interviews by summarizing the texts and identifying key issues that were then labelled as categories. Then, main and sub-categories were attributed to text passages. This “coding” was conducted by each researcher individually using MAXQDA 11 plus (Verbi Software Comp., Berlin, Germany). This software helps researchers to structure and systematically analyse large amounts of qualitative data. The coding was then discussed in the interdisciplinary research team, which consisted of two dentists and two sociologists, to achieve intersubjective validity of the results. Finally, passages of each subcategory were summarized under common themes.

## Results

The results are divided into Dentists' perspective and GPs' perspective, and further subdivided into their expectations, experiences and self-perception.

### Dentists’ perspective

#### Dentists’ expectations of GPs in interprofessional collaboration

Dentists expressed several expectations with regard to interprofessional collaboration with GPs, which will be described below.

##### Dentists’ expectations of GPs’ general professional role

According to dentists, there is a special relationship between GPs and patients. GPs receive the highest confidence from their patients. Thus, GPs are the first contact for patients in case of illness, and patients’ inhibitions about visiting a GP are low. Because of this special relationship between GPs and patients, dentists believe that the primary responsibility for the diagnosis of diseases rests with the GP. Thus, GPs are expected to know their patients’ health status and have all information about their patients’ medical history, treatments and medication.

*DP-M3: “Well that is simple… because the GP is a kind of a confidant [for the patient].”*

##### Dentists’ expectations of GPs’ knowledge of oral health issues

Most dentists expect GPs to have basic dental knowledge covering common oral diseases (e.g., gingivitis/periodontitis, caries, dental erosions, leukoplakia/carcinoma of the soft tissues). In addition, GPs should be able to roughly diagnose these common oral diseases on sight or through a simple examination (i.e., as a tentative diagnosis). One dentist stated that GPs should be particularly able to diagnose halitosis.

*DP-M3 “[…] especially halitosis; some periodontal diseases are accompanied by a typical smell. Well, you can already smell it right when the patient walks in, and if the GP looks into the mouth, then you could see it [the periodontal disease].”*

*DP-G3 “[…] sometimes you think – the GP actually should have noticed this bad breath, too […] if the GP comes close to the patient, says ‘please cough’ and nearly slumps due to the odour […]. Then, it would be probably good not only to refer to the stomach or body hygiene but also to say, ‘Have your gums ever been checked?’”*

Additionally, most dentists expressed the expectation that GPs should be informed about interactions between systemic and oral diseases and, as a consequence, should develop “oral health awareness.”

##### Dentists’ expectations of GPs’ knowledge of medication and side effects for oral health

Most dentists stated that GPs should be well informed about the side effects of medication, especially side effects that can affect oral health, as well as drug interactions. In this regard, anticoagulants and bisphosphonates are of particular concern. In addition, several dentists expressed that they expected GPs to possess knowledge about their patients’ medication and to be able to quickly and competently provide information on this issue to dentists upon request.

##### Dentists’ expectations of GPs’ action for oral health

In line with their expectation that GPs should have knowledge about common oral diseases and the consequences of systemic diseases such as diabetes for oral health, most dentists stated that they expect GPs to inspect the oral cavity (soft tissues, teeth, prostheses) as part of routine examinations and to advise patients to see a dentist if they suspect an oral disease. At a minimum, dentists expect to be considered when the GP detects halitosis in a patient.

*DP-G3: “Well, the connection between diabetes … CHD, periodontitis …, this [awareness] would be desirable, yes, but I guess the doctors, the human medical doctors, are overchallenged with this. At least at the moment.”*

*DP-L3: “Well, one knows that periodontitis is associated with other diseases, such as stroke, heart, risk of diabetes, etc. I guess, in that respect, one could collaborate better, I think.*

##### Dentists’ expectations of GPs’ attitude towards cooperation with dentists

In general, dentists expected GPs to have an interest in establishing good collaboration with dentists to their patients’ benefit. One dentist even expected that GPs should establish contact with a residential dentist when starting their office practice.

*DP-M3: “Well, I would expect that a [newly registered] GP gets in touch with me [laughs] and introduces himself.”*

Several dentists stated that they expect GPs to have sympathy for dentists’ queries regarding their patients’ medication, particularly anticoagulants, or general oral health issues and to be willing to give binding and concisely written information about patients’ general health conditions, medication and treatments. For this purpose, several dentists expressed their desire for GPs to be easily accessible by telephone.

*DP-G4: “For me it is always important - to begin with - that the GP can be reached, and actually, that he can be reached straightaway.”*

### Dentists’ experience of interprofessional collaboration with GPs

#### Dentists’ experience of GPs’ general professional role

Most dentists reported that they experience GPs as competent and as taking good care of patients. Additionally, they felt that GPs know their patients’ general health status and attempt to diagnose diseases such as diabetes early in the course of illness.

*DP-M1: “In my [predominantly old] patients, […] periodontal diseases are very frequent. Thus, further secondary diseases are very frequent, too. However, about all that I do not have to tell anyone the story – the patients are obviously under GPs’ surveillance […] GPs are really meticulous about not accidentally overlooking adult-onset diabetes.”*

By contrast, some dentists expressed dissatisfaction with GPs’ general medical expertise and commitment to patient care. One dentist expressed doubts about GPs’ general medical expertise and treatment of patients.

*DP-G1: “The GP should do his job properly; and on this point, they already fail quite often (laughs).”*

In addition, two dentists noted that GPs may have only noticed patients’ incipient dementia upon receiving an indication from dentists. Additionally, according to some dentists, GPs do not sufficiently inform their patients about their medication. Finally, GPs’ commitment to care for patients living in nursing homes for the elderly was assessed as unsatisfactory by some dentists.

*DP-G4: “Sometimes I have the feeling that the retirement home is not looked after one hundred percent by the supervising GP as it should be. Strictly speaking, about the dry mouth, or nutrition, or drinking patterns… the GPs could do slightly more.”*

##### Dentists’ experience of GPs’ knowledge of oral health issues

Most dentists reported that they experienced the majority of GPs as having virtually no or only very limited knowledge about oral health (e.g., dental causes of general diseases, diseases in the oral cavity and the head region, and particularly temporomandibular disorders (TMD)). This situation becomes clear when GPs refer patients with symptoms of possible stomatognathological origin (e.g., dental or masticatory causes of pain, idiopathic headache, tinnitus) to various specialists but not to the dentist.

*DP-M1: “Regarding the wide field of TMD [and oro-facial pain], no one – not even specialists including ENT doctors – thinks about the dentist.”*

According to some dentists, they find that GPs not only lack knowledge about dental diseases but also lack a general understanding of dental diseases. Several dentists claimed that there is no point in trying to explain to GPs the difference between different dental diseases.

*DP-G3: “If you try to explain the difference between pulpitis and gingivitis to a GP, this is almost … almost impossible.”*

Finally, according to the dentists’ experience, only a few GPs are informed about the interaction between systemic diseases such as diabetes and oral health and thus do not take action.

*DP-M5: “Well, OK, in terms of diabetes for instance, it is accompanied by periodontitis – or the other way around. This results also in difficult [diabetes] control. You can notice this more and more, but very little [initiative] comes from the GPs.”*

*Interviewer: “Was there a GP who asked you to check for periodontitis because the patient suffered from diabetes, for instance?”*

*DP-L3: “I do not know one, no.”*

Some dentists suspect personal disinterest and a lack of education to be the causes of GPs’ knowledge gaps with regard to oral health.

*DP-G4: “The problem is that, unfortunately, the GPs have nearly no clue about dentistry. During their studies, this very lecture [on dentistry] is either not attended or quickly forgotten, because it is a rather short story—dentistry within general medicine […] They know a mite, that there is something [dental], but they are ignorant of how it is connected, of the linkages [between general and oral health].”*

##### Dentists’ experience of GPs’ knowledge of medication

Several dentists reported that they experience GPs as well informed about medication, including anticoagulants, and as providing the right medication to their patients, including medications for the control of diabetes. One dentist reported that GPs are better informed about medication, particularly bisphosphonates, than they were in the past.

*DP-L2: “Concerning bisphosphonates, I can tell that … increasingly over the last five years, patients are well informed by their GPs and specialists.”*

By contrast, several other dentists felt that GPs had no or only very limited knowledge about the dental side effects of drugs.

*DP-M1: “They [GPs] do not have any knowledge about special problems. They do not know what they prescribe and which consequences arise in dentistry. They are completely surprised to poach into dental medicine.”*

This lack of knowledge may involve antihypertensive and psychotropic drugs:

*DP-M3: “[…] hypertensive drugs, which are the rule in an elderly clientele, and the side effects, which encompass hyperplasia [of the gingiva] some of the time—most of the GPs do not know even a little about.”*

This lack of knowledge may also involve bisphosphonates and anticoagulant therapy. Several dentists complained that GPs did not know the relevance of an interruption or bridging of anticoagulant therapy or the use of antibiotic prophylaxis for patients who were to receive dental treatment. Some dentists also reported that GPs were not informed about the newest guidelines on coping with anticoagulation before dental treatments.

*DP-L2: “For reasons of safety, we actually often contact [the GP]. That is always interesting. For a long time, you mustn’t stop the intake of [acetylsalicyl acid], then you should stop, and now for about four years you needn’t stop for little interventions. […] and time and again if you call GPs, they say, ‘Absolutely stop the intake,’ and you answer, ‘No, it is not necessary,’ and then they say, ‘Oh, I did not know that.’”*

However, some dentists believe that GPs are informed about the relationship between bisphosphonates and necrotic changes of the jaw (BRONJ) but not about the course of the consequent potential oral diseases.

##### Dentists’ experience of GPs’ action for oral health

Some dentists are satisfied with the commitment of GPs to dental care and the overall interprofessional collaboration with GPs.

They report that GPs refer patients to them and believe that GPs have sympathy for dentists’ questions concerning patients’ medication. Communication with GPs is assessed as working well. Several dentists also reported that they have professional exchanges with GPs they know personally or that they have close collaboration with GPs concerning the referral of common patients to specialists.

By contrast, most dentists are not content with GPs’ action for oral health. They stated that they suspect that GPs, with the exception of some “medically attentive colleagues,” seldom inspect patients’ mouths or concentrate on the pharynx and tonsils only. They feel that GPs advise patients to see a dentist too late and often do not even send patients with apparent dental disorders to a dentist. One dentist reported that GPs even try to treat dental diseases themselves.

*DP-L1: “Swellings in the mouth are obviously of odontogenic cause to me. However, it is often the case that they tamper with them for several weeks before referring the patient to me.”*

Additionally, dentists state that GPs do not send patients who receive medication with possible dental side effects, such as bisphosphonates, or patients with diabetes to dentists to control their dental status.

*DP-L3: “It would surely be desirable…I think the diabetes is treated and no one thinks about the possible referral to the dentist because there are problems – indeed!”*

The same experience is highlighted with regard to patients suffering from systemic diseases that impact oral health.

*DP-G4: “In my opinion, a patient with diabetes belongs also in [my] practice to check up […] However, these patients are seldom sent to us.”*

##### Dentists’ experience of GPs’ attitude towards interprofessional collaboration with dentists

In general, some dentists experience GPs as lacking dental awareness and a basic willingness to cooperate.

*DP-L4: “Well, actually yes, so…to be honest, I would wish for more cooperation. Recently, I tried to gather information about a patient, and the colleague [GP] stated that it was exaggerated what I was doing.”*

Several dentists reported that they experience collaboration with GPs as frustrating because GPs are contacted only with difficulty and show a lack of sympathy and comprehension for dentists’ questions. With regard to professional exchanges, dentists report that these rarely occur because of overwork. Dentists cite a lack of time and financial compensation for actions as barriers to more intensive collaboration.

However, some dentists express understanding of GPs lack of time, which they perceive to be a consequence of basic conditions established by the healthcare system.

*DP-L4: “I have sympathy for my colleagues because they are hard-pressed for time. In my opinion, there are still not enough GPs, and they all have to somehow treat their patients. That is the problem.”*

#### Dentists’ perception of their role in collaboration with GPs

Most dentists stated that they are informed about drug interactions and side effects as well as the latest guidelines on anticoagulant therapy.

*DP-G2: “The [patients] bring their list [of medication], and of course we do see whether we should better contact the GP or not prior to certain [dental/surgical] procedures.”*

However, a few dentists admitted that they are not well informed and must check with GPs on these issues.

*DP-M1: “The lists of medication vary widely in my patients. You always have to update these, which is not that easy for a dentist […] because the changes are quite frequent. Consequently, the medication has to be explained to me by the patient quite often, what it is and how it works because, as I said, I do not know everything about the drugs that are on the market.”*

*DP-G3: “A major problem, which has nothing to do with the GPs, is that we [dentists] have no clue about what [medication] the patients take. […] Two or three [out of the taken medications] interfere with my treatments, but if it is ultimately a badly adjusted diabetes or, in fact, an anticoagulant or whatever, [we do not know].”*

Several dentists stated that they engage in interprofessional collaboration with GPs. However, cooperation seldom goes beyond occasion-related contact. Dentists reported that they initiated contact with GPs on several occasions, namely, pharmacologic interactions and side effects, planned oral surgery, inflammation within the jaw bones, dementia, or their need for a report of the general medical situation. Thus, dentists claim expertise in detecting these general diseases. Some dentists also report examples of diseases they have diagnosed, such as diabetes, acromegaly, and cardiac insufficiency.

One dentist reported that he has cooperated with a GP for the psychiatric hospitalization of a patient. Another dentist reported starting cooperation with GPs in cases where he detected patients’ lack of compliance with medication.

Some dentists also reported that they strive for knowledge exchange with GPs in regular meetings. However, engagement and exchange are limited by time constraints.

Several dentists reported that collaboration works better with GPs who are personally known to them.

*DP-M3: “Well, there are GPs who would rather send [patients] to me … this might be based on private contact, in fact. However, I am actually … not [in contact] with all, but many [GPs] around me.”*

### GPs’ perspective

GPs also reported several expectations and experiences of collaboration with dentists, as will be discussed below.

### GPs’ expectations of dentists in interprofessional collaboration

#### GPs’ expectations of dentists’ professional role in cooperation with GPs

Several GPs stated that they perceive dentistry and general medicine to be two separate disciplines.

*GP-M3: “A dentist and a GP are two stories that lie far apart.”*

*GP-G4: “I assume that these are two different subjects [dental medicine and general medicine], which are arranged parallel and do not belong together. […] I also guess, but I am not sure, the dentists actually work for themselves [working on their own].”*

Thus, GPs perceive dentists to be quite distant from them and do not expect much collaboration. Dentists are thus only expected to treat their patients’ oral diseases properly; they are not perceived as capable of the early detection of systemic diseases.

*GP-M2: “Now especially, regarding particular diseases, which I find … it would make sense to me that a dentist primarily looks at…no, I cannot think of any.”*

*GP-L4: [hesitates] “Actually…I cannot imagine any situation where I could say, [the patient] has been perpetually at the dentist, who quasi is also a colleague, who could have known, must have seen [detected] this [disease]. No, I have no idea.”*

Only one GP who pursued a holistic approach to health saw dentists as a partners and perceived oral health as part of primary care.

*GP-M1: “Every now and then, the urologist, the dentist…that is part of the ‘check-up package’ […] because teeth actually go with it, somehow.”*

##### GPs’ expectations of dentists’ knowledge of general health issues and medication

In line with the expectations of dentists’ professional role, GPs’ expectations with regard to dentists’ general medical knowledge diverge.

Because they perceive dentistry and general medicine as two separate disciplines, most GPs do not expect dentists to have much knowledge of general health issues. Consequently, and as shown above, GPs do not expect dentists to be able to diagnose any systemic or general disease of their patients, except simple strep throats and skin alterations. This expectation is connected to a low expectation regarding medication and drug interactions.

*GP-L1: “Speaking about medication, [dentists] are too specialized within their subject. There is one antibiotic, and there is paracetamol. I guess besides clindamycin and paracetamol, dentists do not know any other medications. […] And if the dentist does not prescribe drugs, one does not have to know interactions, and both of the mentioned drugs do not have many interactions with other medications.”*

However, individual GPs expect dentists to be able to diagnose the following diseases: craniomandibular dysfunction in connection with cervical syndrome, necrosis caused by bisphosphonates or anaemia, diabetes due to symptoms of increased imbibition and polyuria, and intraoral leukoplakia or extra-oral carcinomas.

Additionally, several GPs expressed their wish that dentists would be well versed in medication, particularly bisphosphonates and anticoagulants.

##### GPs’ expectations of dentists’ action for general health

Most GPs expect dentists to stick to the treatment of oral diseases as any other disease is already treated by GPs themselves, who are consulted by patients more often than dentists are.

*GP-M6: “I have never experienced that a dentist says, ‘You should see a GP about this” because [dentists] treat what they have to treat; all other diseases are treated here [by the GP].”*

*GP-G1: “People prefer to see the GP rather than the dentist.”*

*GP-G4: “Young patients visit the dentist less often than the GP because they have better teeth.”*

As some GPs do not see a connection between dentists’ work and their own work, they also do not expect dentists to report their findings in written form to them and are not even interested in these reports. GP-M3 was *“not very interested about which filling on which tooth has been fixed…”* and stated the following:

*GP-M3: “[Reports from the dentist] would imply that even more letters which I have to address would be delivered here every day. I think this would […] go beyond the scope [of collaboration].”*

*GP-G2: “[Reports from the dentist] are not necessary because these would have no consequence [for the GP].”*

Some GPs also expressed their wish that dentists would not interfere in GPs’ treatment and medication of their patients.

*GP-L2:“Well, as a rule, I do not intervene in the dental treatment, and my colleagues [dentists] normally do not intervene in my treatments […]. One tries to at least adapt to each other in a way that what one is doing is not contradictory to what the other wants to do.”*

However, several GPs stated that they expect to be contacted by dentists after sending a patient to a dentist as well as in case of planned dental treatments, such as tooth extraction. GP-G2 expressed the wish *“that the dentist simply calls me after [I] sent a patient.”*

In the latter case, dentists should also ask about patients’ medication and how to address it.

### GPs’ experience of interprofessional collaboration with dentists

#### GPs’ experience of dentists’ knowledge of general health issues and medication

Most GPs agree that, in general, dentists treat their patients’ oral diseases competently.

*GP-M6: “Every dentist treats [the patients] the way it should be […].”*

By contrast, only one GP reported that he has had the experience that dentists are overchallenged with a proper periodontitis treatment. One GP stated that according to his experience, dentists have expertise about cervical diseases that other doctors lack.

However, many GPs reported that they experienced dentists to have little general medical knowledge, which they attribute to dentists’ specialization and the separate education of dentists and GPs in Germany. Additionally, most GPs report that, according to their experience, dentists are not well informed about medication interactions and side effects and do not know the latest guidelines on anticoagulant therapy.

*GP-G2: “There are clear guidelines [for patients under anticoagulation therapy], but I have the impression that dentists have not read these guidelines thoroughly.”*

Hence, dentists do not know how to deal properly with patients in need of antibiotic prophylaxis or who take anticoagulants if they require dental treatment, such as a tooth extraction. GPs remarked that dentists often tell patients to stop taking their medication without checkingwith the GP, which GPs perceive as interference with their treatment and strongly object to.

However, several GPs reported positive experiences of being contacted by dentists who checked with them about how to address anticoagulants or bisphosphonates.

##### GPs’ experience of dentists’ attitude towards cooperation with GPs

Most GPs have experienced collaboration with dentists as satisfying. If GPs send their patients to a dentist, the dentist examines the patient promptly and is willing to find the causes of oral health problems.

*GP-L3: “[There is a] perfect collaboration…because the colleague promptly sees the patient. This is the most important thing.”*

Additionally, dentists are willing to report their findings to GPs.

Collaboration is reported to work best if the GP and dentist know each other personally.

*GP-M4: “It is not onerous, it is quite good, because we are in personal contact. Thus, you always get more [information] because …the dentist has … no objections to tell you something when calling you.”*

*GP-M5: “It is extremely fascinating, an acquaintance – she is a dentist – if she calls and says, ‘I have a problem.’ She actually directly calls in here, sitting next to the dental chair, and says, ‘Hey, listen, so and so, this and that’…She has no office nearby, somewhere around or near Littletown, but she calls. Then we can exchange views about antibiotics or something else – this is really an enrichment. For me, every exchange is an enrichment.”*

*GP-L3: “We used to live next door, and we were on good terms, and it became a kind of tradition [that I call the dentist].”*

#### GPs’ self-perception of their role in collaboration with dentists

Most GPs do not see a need for collaboration with dentists and seldom contact dentists. Many GPs report that they perform an oral inspection as part of routine diagnosis. If, in doing so, they diagnose a desolate dental status (carious lesions, missing/fractured teeth) or inflammation of the oral soft tissues (periodontitis, candida), they see themselves as responsible for sending a patient to the correct specialist.

*GP-G2: “Every two years, I do the health check-up in patients aged 35 and older. I also have a thorough look at the teeth – as far as I can judge it – and advise sometimes: ‘This tooth has no antagonist or something; you have to see the dentist’ […].”*

The few GPs who followed holistic approaches in their daily practice included halitosis, pain of the jaw and face as well as chronic diseases as causes for a referral to the dentist.

In addition, most GPs state that they are informed about the consequences of diabetes for oral health as well as the side-effects of medication, especially bisphosphonates and anticoagulants, for oral health. However, they do not derive a need for action from this knowledge.

Furthermore, some GPs perceive themselves as the responsible contact person for queries about their patients’ medication. This is why they see themselves as primarily responsible for the adaptation of anticoagulation therapy if a dental treatment is planned.

*GP-L2: “OK, normally, the patient is treated by both [GP and dentist]. Thus, it is up to the dentist to check back routinely concerning medication - anticoagulation foremost – or that the dentist is asking in fact for analgesics, [or] if someone suffers from CHD for instance – I am inculcating these very [CHD] patients to talk it over [with the dentist]. Sometimes there are also queries concerning antibiotics.”*

## Discussion

### Key findings

The findings show discrepancies between GPs’ and dentists’ expectations, experiences and self-perceived roles in interprofessional collaboration (see Tables 3 and 4). On the one hand, GPs fall short of dentists’ expectations with regard to GPs’ dental knowledge, their awareness of oral health as well as their commitment to intense collaboration. However, dentists also mentioned exceptions to this rule.

**Table 3:** GPs’ and dentists’ expectations and experiences of collaboration (summary)

Dentists’ expectations of collaboration with GPs	Dentists’ experiences of collaboration with GPs
GPs should …	GPs …
•… know their patients’ health status, treatment and medication and be able and willing to pass this information •… have basic dental knowledge and be informed about interactions between systemic diseases and oral health •… be able to diagnose common oral diseases •… be informed about medication and its side effects for oral health •… inspect the oral cavity of their patients as part of their routine •… be interested in establishing good cooperation with dentists •… have sympathy for dentists’ queries and be easily accessible	•… are mostly experienced as competent and taking good care of their patients •… strive to diagnose illnesses early •… surveil diabetes, etc. rigorously •… mostly lack basic dental knowledge and oral health awareness •…mostly are not informed about interactions between systemic diseases and oral health •… mostly do not inspect the oral cavity as part of routine examinations •…do not inform their patients sufficiently about their medication side effects on dentistry/oral health (including anticoagulation) •… are not sufficiently committed to patients living in nursing homes •… are not sufficiently committed to collaboration with dentists
GPs’ expectations of collaboration with dentists	GPs’ experiences of collaboration with dentists
Dentists should…	Dentists …
•… not necessarily be able to diagnose general or systemic diseases •… stick to the treatment of oral diseases as all others are treated by GPs •… treat oral diseases/teeth competently •… be well-versed with medication, particularly bisphosphonates and anticoagulants. •… check with GPs with regard to their patients’ medication before dental treatment and not intervene in medication •… according to a single opinion, report dental treatment to GP	•… have limited general medical knowledge •… treat their patients competently •… are not well informed about medication and do not know guidelines on anticoagulant therapy •… mostly do not check with GPs with regard to medication before dental treatment and intervene in medication •… examine patients and informing GPs about dental treatment upon request •… are willing to collaborate with GPs

**Table 4:** Self-perceptions of Dentists and GPs (summary)

Dentists self-perception	GPs’ self-perception
Dentists …	GPs …
•… are informed about drug interactions and side effects •… are mostly aware of latest guidelines on anticoagulant therapy •… initiate contact with GPs on several occasions •… are interested in knowledge exchange with GPs but lacking time •… are committed to collaboration with GPs	•… routinely examine dental status •… send patients to a dentist if they diagnose poor dental status •… are aware of side effects of medication •… are responsible for patients’ treatment and medication •… are informed about consequences of diabetes for oral health

On the other hand, dentists fall short of GPs’ expectations with regard to dentists’ knowledge about medication, particularly bisphosphonates and anticoagulants, as well as their willingness to check with GPs with regard to their patients’ medication before dental treatment. In summary, GPs rarely see occasions for more collaboration and interprofessional patient care with dentists. Both professions perceive their own role and action for their patients’ health more positively than the other does. The exceptions to the rule highlight the impact of mutual perceptions, which can bridge the administrative separation in daily practice.

Nevertheless, it is notable that the socio-economic/social aspects of the patients were not thematised by both groups. This is interesting because both types of doctors have high patient loyalty, with patients returning for years, and GPs address this aspect as a focus in their daily practice [31]. In this context, one female GP noted the prevalence of psychosomatic disorders and thought about the presentation of symptomatic patients in the dental office, resulting in a need for communication to prevent polypragmatism.

### Strengths and limitations

This study is the first to evaluate GPs’ and dentists’ expectations and experiences of interprofessional collaboration. The qualitative method was well suited to investigate this topic as it allowed us to capture motivational factors for collaboration as freely expressed by the participants and provided rich material. A further strength of the study is that analysis was conducted by researchers with different backgrounds (dentists, sociologists), thereby increasing intersubjective traceability.

Although a qualitative study cannot claim representativeness, a possible limitation of the study may be that, because of difficulties in recruiting participants for the study, the sample was not balanced with regard to gender and age. First, considerably fewer female than male GPs and, in particular, fewer female than male dentists participated in the study for unknown reasons. However, the statements of participating male and female GPs did not differ; this finding was also reported by Probst et al. [18] with regard to GPs and specialists. Thus, it is uncertain whether a new perspective would have been added by a more gender-balanced sample. Second, the mean age for participants was above 50, implying that the perspectives of young professionals, which might differ from those of older professionals, were missing [32, 33]. These potential differences in perspectives could be explored by further research.

A further possible limitation of the study may be that the study was undertaken in one region of Germany; thus, the findings may not be directly transferable to other countries with a separation of dentistry and general medicine. Additionally, as the study was conducted in only one region of Germany, it is uncertain whether the results are transferable to regions where dentists and physicians are or have been educated together (e.g., in Eastern Germany during the German Democratic Republic) in a so-called “stomatological curriculum” [34, 35].

### Comparison with existing literature

The finding that interprofessional collaboration between dentists and GPs is hindered by a perceived distance between both professions is in line with the findings of a recent study on the interface of GPs and dentists in primary care [24] and confirms the same story of “parallel universes” [3, 36].

International research has also found perceived distance to hinder cooperation between GPs and specialists [31, 37, 38] as well as between GPs and occupational health physicians [39, 40]. Thus, the impact of common socialization during academic education on later cooperation between professions during their working career is emphasized [22].

### Implications of findings

In addition to university curricula, joint postgraduate continuing education is needed [41–43] to allow both knowledge transfer and networking of the two professions to overcome established structures of interaction [44, 45]. This can be facilitated through joint programmes of the two professional bodies (chambers).

All aspects addressed here involve tremendous tasks to improve the interface and interaction between GPs and dentists, which has recently been addressed in an entire issue of the Dental Clinics of North America [46] and the Horizon 2020-funded ADVOCATE project [47]. Above all, it remains questionable how improvements in cooperation and interprofessional collaborative practice could be measured within a health system that does not regard dental medicine as a transferring doctor or as a medical specialist [7, 27]. The broad range of this international literature, which calls for more interprofessional collaboration, shows that the separation of professions is persistent and is not specifically a German problem [3, 46, 48, 49]. Thus, policymakers should implement the recommended changes to the system [50].

### Recommendations for further research

The results imply a need for further research on feasible approaches to interprofessional education in health systems where medical and dental care are organizationally separated [25]. Furthermore, investigation of the impact of the feminization of medicine and the joint education of dental and medical students on the later collaboration of these professionals is required. It might also be of interest to examine patients experience and judge the cooperation of dentists and GPs.

## Conclusion

The results show that collaboration between GPs and dentists is not very intensive and is largely of a therapeutic rather than preventive or holistic character, as would be required in light of evidence of connections between oral and systemic diseases. More profound and regular collaboration is apparently hindered by a perceived distance between dentists and GPs as well as a lack of knowledge on both sides that may result from the separate academic education of dentists and GPs. Thus, to improve interprofessional collaboration, under- and postgraduate curricula of dentistry and medicine could be updated, and more joint education could be introduced to promote interprofessional collaborative practice.

## Abbreviations

ÄK: Chamber of phycisians (regulatory association for professional self-government)
BRONJ: Bisphosphonate-related osteonecrosis of jaw
CHD: Coronary heart disease
DM: Studies of dentistry (dental medicine)
DMP: Disease management program
DP: Dental practitioner (dentist)
GP: General practitioner
HM: Studies of (human) medicine
KV: Association of SHI physicians (professional body)
KZV: Association of SHI dentists (professional body)
SHI: Statutory health insurance
SPSS method: “Collect, Check, Sort, and Subsume method” to create guiding questions
TMD: Temporomandibular disorder
ZK: Chamber of dentists (regulatory association for professional self-government)
